# Optimization and Modeling of Material Removal Rate in Wire-EDM of Silicon Particle Reinforced Al6061 Composite

**DOI:** 10.3390/ma14216420

**Published:** 2021-10-26

**Authors:** Deepak Doreswamy, Anupkumar M. Bongale, Marcin Piekarski, Arunkumar Bongale, Satish Kumar, Danil Yurievich Pimenov, Khaled Giasin, Krzysztof Nadolny

**Affiliations:** 1 Department of Mechatronics Engineering, Manipal Institute of Technology, Manipal Academy of Higher Education, Manipal 576104, India; 2 Department of Computer Science and Information Technology, Symbiosis Institute of Technology, Symbiosis International (Deemed University), Lavale, Pune 412115, Maharashtra, India; 3 Mathematic and Physics Department, Gdansk University of Technology, G. Narutowicza 11/12, 80-233 Gdansk, Poland; piekarz2121@gmail.com; 4 Department of Mechanical Engineering, Symbiosis Institute of Technology, Symbiosis International (Deemed University), Lavale, Pune 412115, Maharashtra, India; arunbongale1980@gmail.com (A.B.); satishkumar.vc@gmail.com (S.K.); 5 Symbiosis Centre for Applied Artificial Intelligence, Symbiosis International (Deemed university), Lavale, Pune 412115, Maharashtra, India; 6 Department of Automated Mechanical Engineering, South Ural State University, Lenin Prosp. 76, 454080 Chelyabinsk, Russia; danil_u@rambler.ru; 7 School of Mechanical and Design Engineering, University of Portsmouth, Portsmouth PO1 3DJ, UK; khaled.giasin@port.ac.uk; 8 Department of Production Engineering, Faculty of Mechanical Engineering, Koszalin University of Technology, Racławicka 15-17, 75-620 Koszalin, Poland; krzysztof.nadolny@tu.koszalin.pl

**Keywords:** Wire-Electrical Discharge Machining (Wire-EDM), material removal rate, wire speed, aluminium composite, silicon carbide particles, taguchi method

## Abstract

The mechanical, physical and interfacial properties of aluminum alloys are improved by reinforcing the silicon carbide particles (SiC_p_). Machinability of such alloys by traditional methods is challenging due to higher tool wear and surface roughness. The objective of research is to investigate the machinability of SiC_p_ reinforced Al6061 composite by Wire-Electrical Discharge Machining (wire-EDM). The effect of wire-EDM parameters namely current (I), pulse-on time (T_on_), wire-speed (W_s_), voltage (I_v_) and pulse-off time (T_off_) on material removal rate (MRR) is investigated and their settings are optimized for achieving the high MRR. The experiments are designed by using Taguchi L_16_ orthogonal arrays. The MRR obtained at different experiments are analyzed using statistical tools. It is observed that all the chosen process parameters showed significant influence of on the MRR with contribution of 27.39%, 22.08%, 21.32%, 15.76% and 12.94% by I, I_v_, T_off_, T_on_ and W_s_, respectively. At optimum settings, the Wire-EDM resulted in MRR of 65.21 mg/min and 62.41 mg/min for samples with 4% and 8% SiC_p._ The results also indicated reinforcing SiC_p_ upto 8% showed marginally low influence on MRR. Microstructural investigation of the cut surface revealed the presence of craters with wave pattern on its surface. The top surface of the crater is featured by the recast layers connecting adjacent craters. Further, the statistical model is developed using linear regression to predict the MRR (𝑅^2^—73.65%) and its predicting accuracy is verified by the confirmation trials. The statistical model is useful for predicting the MRR for different settings of the process parameters. The optimized settings can be used to improve the machining productivity by increasing the MRR while machining of Al6061-SiC_p_ (upto 8 wt. %) alloy by wire-EDM industries.

## 1. Introduction

Wire-WEDM is a non-conventional machining method in which the removal of material occurs by the repetitive sparks produced between the electrode and the work material in the dielectric medium. The circulation of dielectric fluid carries away the molten debris from the machining zone. Wire-EDM is extensively used in the industries for producing the complex contours in the electrically conductive materials such as aluminium, copper, brass, titanium and other alloys. The advantages of wire-EDM over other processes are it can produce thin walls with very small radii of internal corners, minimal burrs, high dimensional accuracy, absence of mechanical stress, low surface roughness, fast processing and cost-effectiveness. Due to low density, the aluminium alloys are popularly used in transport applications (automobiles, aeroplanes and ships) for reducing the fuel consumption and thus carbon emission. The sector-wise usage is as follows: the transportation industry—23%, building and construction—25%, packaging industries—8%, engineering industries—11% and electrical appliances—12% [1]. In general, this metal possesses low density, good malleability, corrosion resistance and electricity conductivity. Aluminium 6000 series material shows excellent mechanical properties and weldability, thus it finds applications in aircraft fittings, electrical fittings and connectors, camera lens mounts, marines fittings and hardware, couplings, hinge pins, pistons, bike frames, valve parts, etc. [2,3].

The properties of aluminium can be further improved by alloying with other materials such as SiC, Al_2_O_3_, MgO, WC, S_i_O_2_, B_4_C, etc. Studies shows that yield strength, ultimate tensile strength, elastic modulus and hardness were improved by the addition of SiC and Al_2_O_3_ particles in aluminium matrix [4]. The addition, Al_2_O_3_, zirconium, cerium and magnesium resulted in decreasing the electrical conductivity of the composite [5]. The age hardened Al6061-SiC_p_ reinforced composites showed the improvement in hardness by approximately 120–145% compared to as-cast alloy at aging temperature range of 100 to 200 °C. The lower temperature aging showed substantial improvement in wear resistance compared to high temperature aging [6]. The addition of grain refiners (Al-5Ti-1B) led to size reduction and also transformed a longer and coarser grain to circular shaped and finer grains which significantly increased the hardness, tensile and wear resistance properties, but decreased the ductility [7]. SiC_p_ are used as reinforcement particles because of its high ratio of strength to weight, thermal stability and wear resistance [8,9]. Investigation of micro-structural and mechanical properties of Al 6061 with SiC_p_ (500 nm) showed the improvements in Young’s modulus, strength and toughness [10]. Cao et al. [11] studied the effects of silicon and tungsten carbide reinforcement in aluminum matrix composites. The strong interfacial bond created between the matrix and the reinforced particles in these composites were found to transfer the load from the matrix and distributed to the reinforcement. This resulted in enhancement of the elastic modulus and the strength of composite. Nair et al. [12] made a review of metallurgy of Al-SiC composites in which the elastic stiffening effects of the SiC whiskers are detailed through continuum mechanics models. The type of reinforcement, its size, shape and quantity were found to significantly influence the mechanical properties [13].

Although aluminium possesses good machinability, the machining of SiC_p_-reinforced aluminium alloys is challenging due to its abrasive nature which lead to rapid tool wear and affecting the surface quality [14,15]. The pull-out of the particle from the matrix and fracture of reinforced particles lead to formation of voids and cavities on the machined surface affecting the surface quality. In this scenario, the wire-EDM is one of the viable non-traditional methods in which the material removal occurs by rapid and repetitive sparks which occur by the electric discharge between the tool and workpiece. This causes the localized heating in the spark gap and the temperature developed is high enough to melt and vaporize the base metal. The dielectric fluid is circulated in the spark gap to control the electrical discharge and carry away the heat generated in machining [16]. The limitation of this process is requirement of electrically conductive work piece. The advantage of wire-EDM includes, machining is independent of mechanical properties of the work material, producing the complex profiles and the non-contact between tool and work piece during the machining. The research trends of wire-EDM mainly focus on scientific investigations of applications and optimization of the process parameters. In recent years, EDM is popularly used for making nozzles for automobiles, miniature hole for turbine blade cooling, biomedical device such as stents, micro-channels for microfluidic devices and the production of various micro-electro-mechanical devices, etc. [17]. The Wire-EDM is also found to be an effective tool for polishing applications of 3D printed parts in which about 80% of surface roughness was reduced [18]. The Wire-EDM polished 3D printed SS316L material showed the elimination the balling pits, voids and porosity. The high t_on_ led to roughness due to prolonged duration of discharge, thus the low t_on_ was encouraged for polishing application by wire-EDM. The negligible amount of wire electrode material deposition was seen on the polished on surface.

A. Pramanik [19] investigated the effect of reinforced SiC_p_ size (0.7–13 μm) in aluminium composites which were prepared by hot extrusion and heated treated (T1). The test samples with the smaller sized SiC_p_ and 0% particles did not exhibit significant difference on the surface quality after wire-EDM. The smaller sized SiC_p_ showed larger kerf width and lower electrode wear compared to test samples reinforced with larger particles size. Furthermore, matrix material was coated on the wire-electrode during the machining. The machining performance was found to be improved by mixing the multiwalled carbon nanotubes (MWCNTs) in the dielectric fluid. MWCNTs (1 g/L) mixed with dielectric fluid enhanced the MRR by 75.42% and reduced the SR by 19.15% in wire-EDM of Nitinol shape memory alloy. In addition, a substantial reduction recast layer thickness and other surface defects were also observed [20]. P Sivaprakasam et al. [21] determined the machinability of A413—9% composite using zinc coated copper wire in wire-EDM. The operating parameters such as voltage, capacitance and feed rate showed significant interaction effect on the kerf-width and surface roughness. The MRR was increased with increase in supply current and duration of pulse-on while machining of SiC/Al composite [22,23,24,25]. When composition of SiC_p_ is increased upto 15% in the matrix phase, the MRR was found to decrease and the further increase in SiC content showed the reverse trend. Agrawal et al. [26] investigated the surface roughness and MRR of AA6025 composite (SiC: 10 wt. % and Al_2_O_3_:10 wt. %). The studies showed that the machining performance was improved by using conductive powder in the dielectric fluid. This is due to reduced strength of dielectric fluid by the conductive particles and increased the spark gap. This phenomenon assisted in ignition process by generating a stable and higher discharge in the spark gap [27]. Sidhu et al. [28] studied the EDM surface properties of 6% SiC/A356.2, 10% SiC-5% quartz/Al and 30% SiC/A359. The surface hardness was found to increase with increase in the density of the reinforced particles. In EDM, significant amount of metal transfer was found to occur from the copper electrode compared to graphite electrode. Dey et al. [29] optimized the wire-EDM parameters for machining of Al6061/ceno-sphere composite. The increase in pulse current and pulse-on time produced the larger craters and high surface waviness. This resulted in poor surface quality and increased electrode wear rate in Al (6351)-SiC-B_4_C Composite [30].

The weight and volume fraction of reinforcing particles affects the distribution of particles in the matrix which further affects the mechanical properties of the composite. The homogenous distribution of reinforced particles leads to uniform load distribution in the composite and hence improve its mechanical properties. However, higher loading of reinforced particles led to agglomeration which resulted in reduction of its mechanical properties due to improper load distribution [31,32]. The tensile strength and hardness were found to increase with the addition of SiC_p_ up to 10 wt. % beyond which these properties were gradually reduced. However, the ductility of the composite decreased with increase in SiC_p_ content [33]. The stir casting of the test samples showed the agglomeration of the SiC_p_ particles in the molten Al6061 matrix for the loading beyond 8 wt. %. Considering this the weight fraction is restricted upto 8 wt. % in this work. Machining of Al-SiC_p_ by traditional methods possess challenges of high tool wear-rate, formation of pits, voids, micro-cracks and fractured reinforcements on the cut surface. These challenges can be addressed by non-conventional machining methods such as wire-EDM. Considering these factors, an attempt is made in this work to investigate the effects of wire-EDM process parameters such as current, pulse-on and pulse-off time, wire speed and voltage on MRR and optimize these settings to improve the productivity in terms of MRR. Although, artificial neural network-based optimization methods such as fuzzy AHP-ARAS [34], genetic algorithm [35], particle swarm, moth-flame and Grass–Hooper optimization [36] methods are more attractive tools of optimization for the experiments with large data sets, for limited experimental trails as in this work, Taguchi and response surface [37] methods are more viable methods. Therefore, this works adopts Taguchi method for optimization of MRR and to develop the regression model to predict the MRR. The model estimates the MRR for different settings of these process parameters.

## 2. Materials and Methods

This section provides the details of specimen preparation and experimental set up used for the investigation, settings of wire-EDM process parameters, design of experiments, measurement of the response parameter and optimization methodology.

### 2.1. Experimental Set Up and Specimen Details

**Figure 1:**
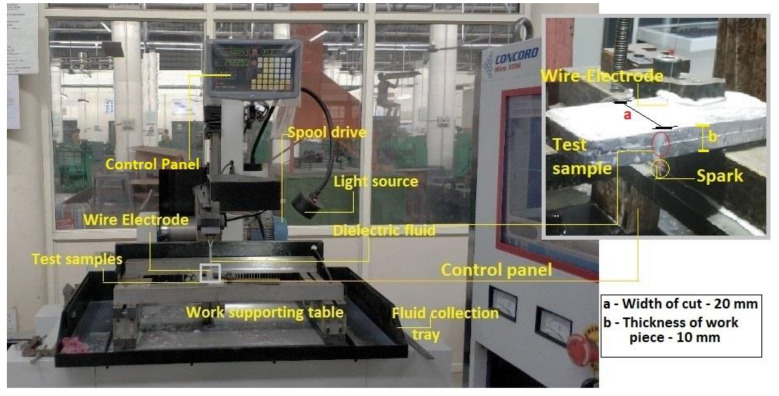
Experimental setup.

Figure 1 shows the details of experimental facility used in the present work. The experiments are performed using a 2-axis (X-320 mm, Y-400 mm) computer numerically controlled wire-EDM made by Concord wire-EDM, Bangalore, India (Model: DK7732). Molybdenum wire (diameter: 0.16 mm) is used tool electrode during the experiments. Mixture of soft water and gel is used as dielectric fluid. The resolution of the controller is 0.001 mm. The SiC particle (size: 170 µm) reinforced Al-6061 alloy is prepared by stir-casting. The matrix material is liquefied at a temperature of 850 °C in a crucible and the pre-heated (650 °C) reinforcing particles (4% and 8% by weight) are added to molten metal and distributed uniformly in the matrix by mechanical stirrer. The liquefied metal is transferred to pre-heated (at 200 °C) mold and then cooled to room temperature. General composition of Al6061 composite is shown in Table 1.

### 2.2. Design of Experiments

The wire-EDM parameters such as current, pulse-on and pulse-off time, wire speed and voltage are chosen to study its effect on MRR. The voltage is varied at two different levels and remaining parameters are varied at four different levels. Table 2 details the wire-EDM process parameters and their chosen settings. These levels are selected based on the test experimentations. Total degree of freedom required for the experimental design is 13, hence experiments are designed using L_16_ (45 × 12) Taguchi orthogonal array. Table 3 shows the experimental design. The experiments were replicated for two trials in each experimental condition. Thickness of specimens (10 mm) and the supply pressure of dielectric fluid were kept constant during experiments.

### 2.3. Measurement of MRR

**Figure 2:**
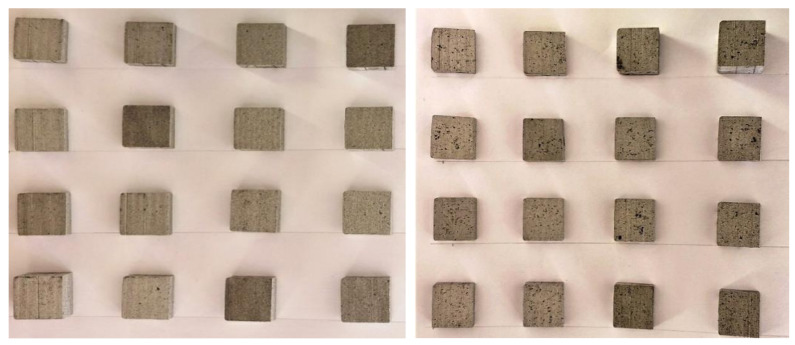
Cut surface of the machined samples.

Test specimens are subjected to wire-EDM for a length of 20 mm according to the design shown in Table 2. MRR obtained for each experimental trial is calculated by weight loss method during the machining as given by the Equation (1). The weight of the specimens before (*w_i_*) and after (*w_f_*) the machining is measured using digital mass balance (accuracy: 0.001 g). For each test samples the measurements were made five times for the accuracy. Table 3 shows the average MRR of each trail for the samples with different composition of SiC_p_. The cut surfaces of the test samples machined at different experimental conditions are shown in Figure 2.

(1) $$MRR=\frac{w_{i}-w_{f}}{t}mg/s$$

### 2.4. Optimization of Material Removal Rate

The experimental results were analyzed by statistical method called Analysis of variance (ANOVA) to identify the effect of Wire-EDM parameters namely current, pulse-on and pulse-off time, wire speed and voltage on MRR. Significant process parameters are identified by conducting F-Test at confidence level of 95%. Further, the average MRR obtained at different settings (Level) is determined. The optimum conditions which generate the high MRR is established based on the levels which produce highest MRR corresponding to each process parameters of the study. The MRR at optimized condition is computed using Equations (2)–(5) [38]. Finally, a statistical model is developed using regression analysis to establish to predict the MRR.

(2) $$n_{eff}=\frac{n}{1+DF}$$

(3) $$T=\frac{\sum MRR}{n}$$

(4) $$MRR_{Optimum}=MRR\left[A_{3}B_{3}C_{1}D_{3}E_{2}\right]-4\times T\;$$

(5) $$\mathrm{Confidence}\;\mathrm{interval}=\pm \sqrt{F\left(\alpha ,DF_{error}\right)\times \frac{MS_{error}}{n_{eff}}}$$

### 2.5. Theoretical Modelling of Material Removal

In Wire-EDM, the material removal occurs through electric discharge in the spark gap. The spark energy developed during the sparking process is determined by Equation (6). The heat produced by the spark depends on current, pulse time and voltage. Although there are different models to predict the spark radius(*r_sp_*), the Gaussian heat input model is popularly used among the researchers [38,39]. According to this model, the maximum heat intensity(*_qR_*) is at the axis of a spark and the corresponding heat flux is given by Equation (7) [39,40]. The electric field produced between electrode and work piece ionize the dielectric fluid which create the path for electrons movement. This lead to formation of spark whose temperature levels can melt and vaporize a tiny volume from the work piece. This phenomenon leads to creation of hemispherical shaped craters on the machined surface and its size can be estimated using Equation (8) [41].

(6) $$E_{s}=I_{p}\times I_{V}\times t_{on}$$

(7) $$q_{f}\left(R\right)=\frac{4.45\times W_{M}\times I\times V}{\Pi \times {\left(r_{sp}\right)}^{2}}\times e^{[-4.5\left(\frac{R}{r_{sp}}{)}^{2}\right]}$$

(8) $$\Gamma =\frac{2}{3}\pi r^{3}$$

## 3. Results and Discussion

### 3.1. The Effect of Current and Wire Speed on MRR

**Figure 3:**
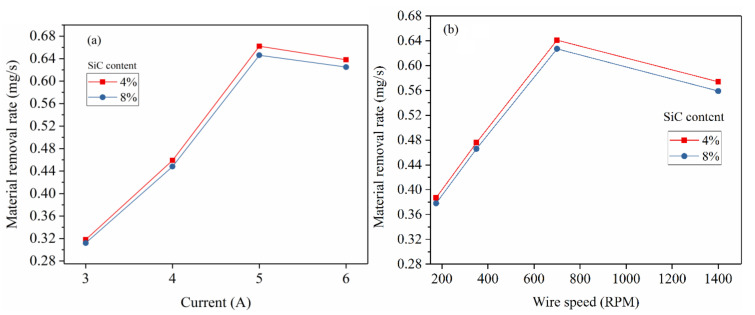
(**a**) The effect of current on MRR. (**b**) The effect of wire speed on MRR.

Figure 3a shows influence of current on MRR. It is observed that, the MRR is increased with increase in current from 3 to 5 A. The maximum MRR of 0.662 mg/s is obtained at current of 5 A. An increase in current level from low to high increase the breakdown voltage and raise its spark energy [36,37] as given by the Equation (6). Due to higher E_s_, the high current results in melting and vaporization of more volume of material [41,42]. Similar trends were also reported by other researchers [43,44]. However, the current beyond 5 A showed a slight decrease in MRR. This may be due to insufficient flushing of molten pool which result in formation of recast layer on the preceding machined surface. Furthermore, increasing the discharge energy beyond the peak levels results in deposition of the debris which contain the decomposed product of dielectric fluid and the eroded particles from the wire-electrode. The current settings beyond the peak levels result in rapid deposition of the debris at the machining gap which leads to significant influence on the characteristics of dielectric breakdown and affects the material removal [45].

Further, Figure 3b shows the influence of wire speed on MRR. The rotational speed of spool on which electrode wire is wrapped is varied from 175 to 1400 RPM and its corresponding MRR obtained is shown in Figure 3b. The MRR is increased from 0.387 mg/s to 0.641 mg/s due to change in spool rotational speed up to 700 RPM. Further increase of spool rotational speed appears to be counterproductive which reduced the MRR by 10.45%. This is attributed to the fact that the amount of spark energy available for machining reduces with increase in wire speed [46]. As a result, the MRR drops for the wire-speed settings beyond the optimum speed.

### 3.2. The Effect of Pulse-On Time and Pulse-Off Time on MRR

**Figure 4:**
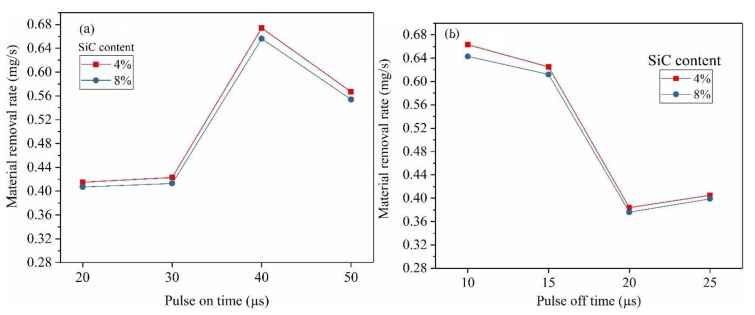
(**a**) The effect of Pulse-on time on MRR. (**b**) The effect of Pulse-off time on MRR.

**Figure 5:**
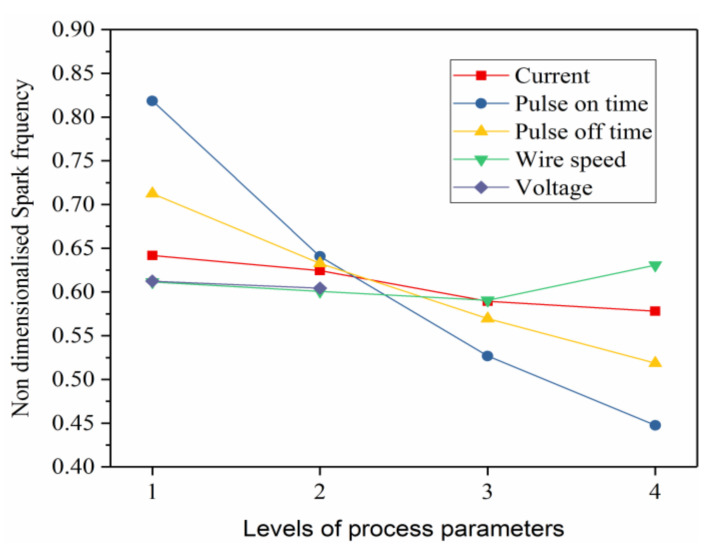
The spark frequency at different levels.

The effect of pulse-on time, pulse-off time on MRR is shown in Figure 4a,b, respectively. It is observed that increase in pulse-on time from 20 µs to 40 increased the MRR. The energy (E) of each spark is given by E = V × I × t_on_, where V—voltage, I—current and pulse-on time. This indicate that the longer spark duration (pulse-on time) releases more spark energy in each spark cycle, resulting in melting of material with larger crater size (i.e., MRR is directly proportional to crater radius). However, the pulse-on time beyond 40 µs showed reduction in MRR from 0.674 mg/s to 0.567 mg/s. This may be due to recasting of the part of molten pool around the craters due to longer cycle time and the reduced spark frequency as seen in Figure 5.

Interestingly, increase in the pulse-off time from 10 µs to 25 µs resulted in steady decrease in MRR as shown in Figure 4b. Pulse-off time is the time duration at which sparking do not occur. Therefore, increasing the pulse of time increase the cycle time and also reduce the spark frequency as shown in Figure 5. This resulted in decreasing the MRR with increase in pulse-off time.

### 3.3. The Effect of Voltage on MRR

**Figure 6:**
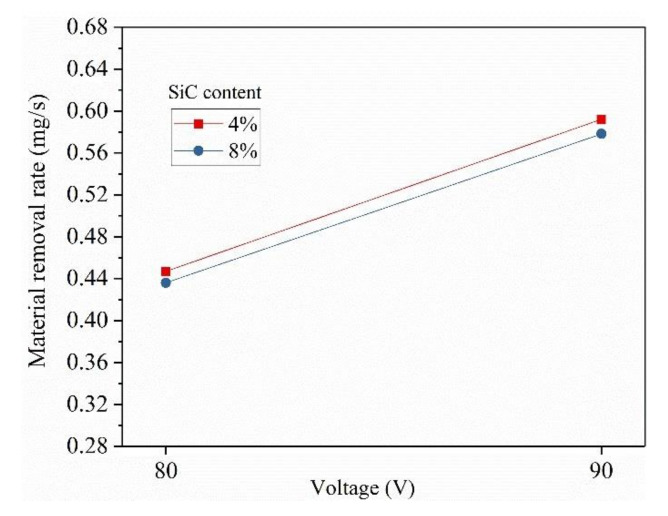
The effect voltage on MRR.

The effect of voltage on MRR is shown in Figure 6. It is observed that MRR is directly proportional to voltage. The MRR is increased by 24.45% with increase voltage from 80 V to 90 V in the spark gap. As explained earlier, the spark energy is a function of voltage, current and pulse-on time (i.e., E = V × I × t_on_). Therefore, the increase in voltage resulted in melting and vaporization of greater volume of material and results in increasing the MRR.

### 3.4. Morphological Study of Machined Surfaces

**Figure 7:**
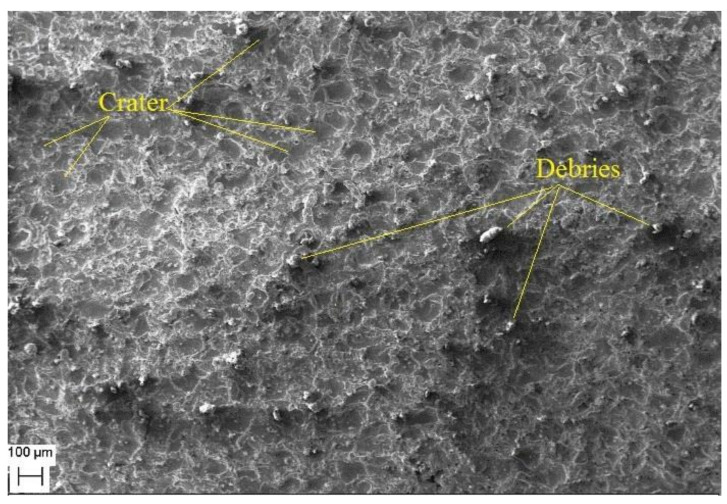
The cut surface.

**Figure 8:**
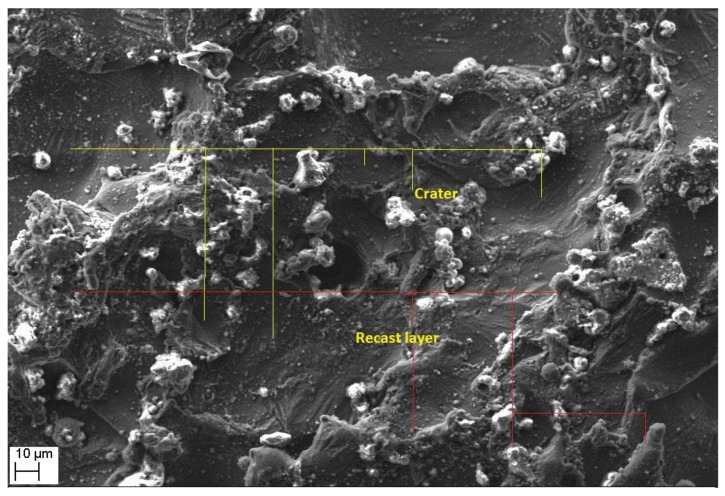
Magnified view of the recast layer and craters.

**Figure 9:**
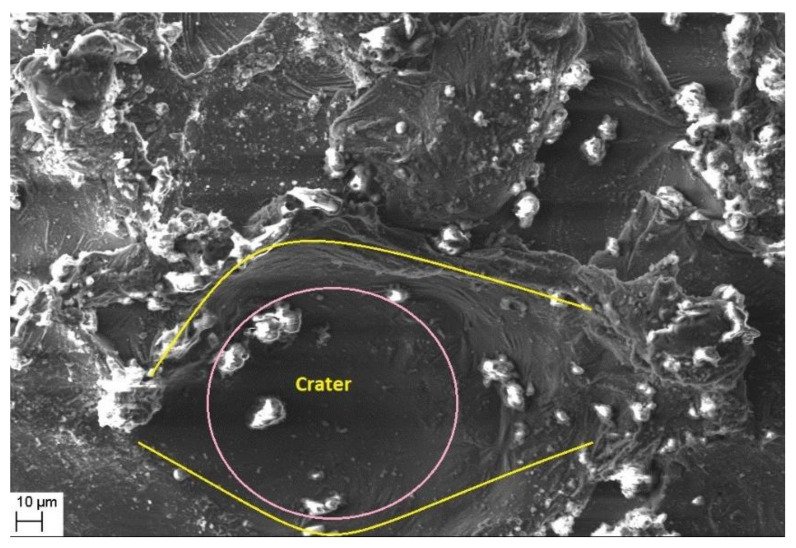
The crater formation.

**Figure 10:**
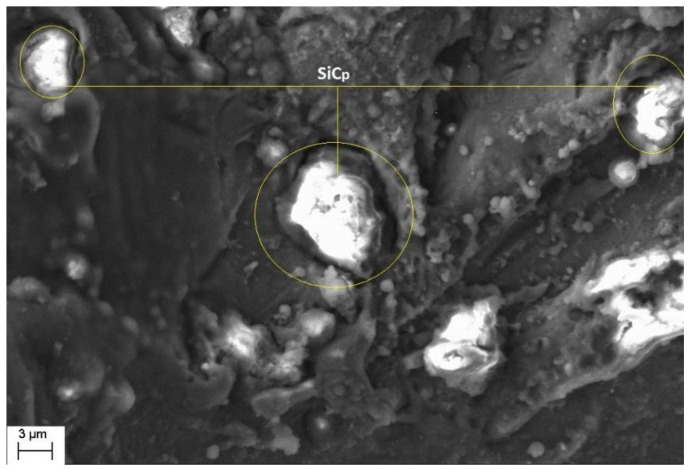
The intact SiC_p_ on the cut surface.

**Figure 11:**
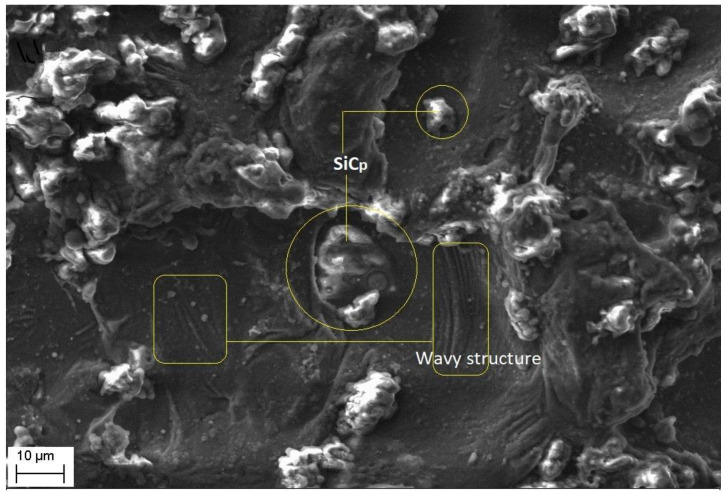
Formation of wavy patter on the crater surface.

**Figure 12:**
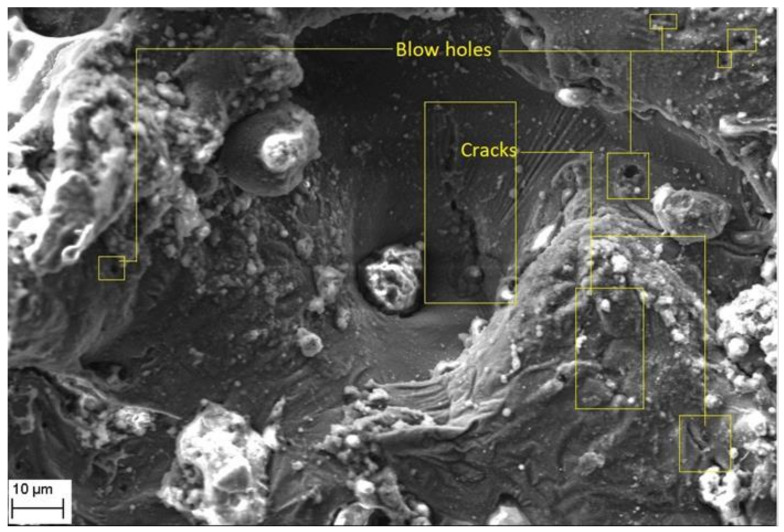
Cracks and blow hole formation on crater surface.

**Figure 13:**
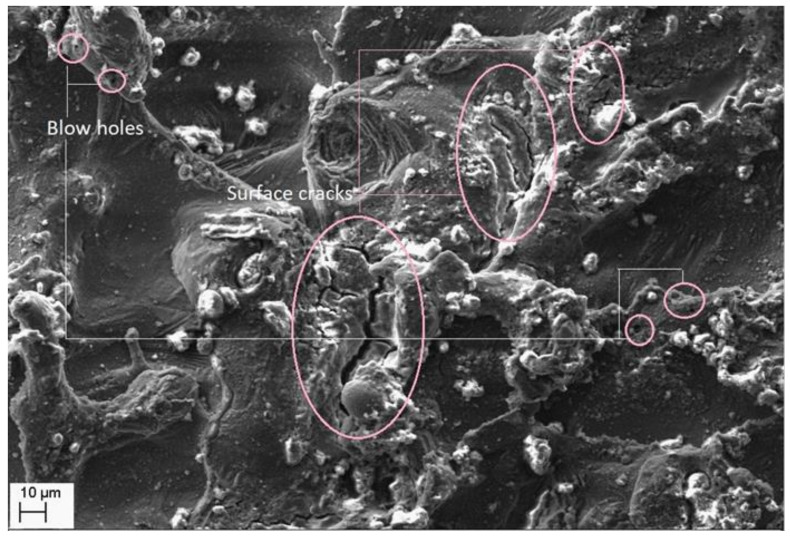
Cracks and voids on the recast layer.

Morphological study of the machined surfaces is carried out using scanning electron microscopy (SEM). Figure 7, Figure 8, Figure 9, Figure 10, Figure 11, Figure 12 and Figure 13 shows the details of microstructural details of cut surfaces. Figure 7 and Figure 8 shows that the machined surface is characterized by the circular shaped craters surrounded by the peaks and valley formed by the re-solidified molten metal pool. The electric spark produced between the wire-electrode and the work piece generates sudden pressure on the dielectric fluid. The series of shockwaves thus formed exert greater pressure on the molten metal pool which lead to formation of craters on the machined surface. The surfaces are also featured by some of the partly molten metal globules which are firmly intact with the machined surfaces.

Magnified views of these craters are shown in Figure 9, Figure 10 and Figure 11. It is observed that the reinforced SiC_p_ particles appear to intact the recast layer and a few particles are found to be relocated on the cut surface. This is due to circulation of the dielectric fluid in the machining process. As the dielectric fluid pass through the machining surface the heated reinforced particles which are in the molten pool are carried along with the flow and such debris gets welded on to the nearby locations on the machined surface during solidification of molten matrix pool. It is also observed from these figures that most of the crater appears confirms to the shape of truncated sphere. This is in confirmation with the Gaussian heat distribution model [39,40] used to predict the spark radius. The boundary of the craters on the machined surface show elongated structure in the direction of wire travel. In fact, the crater surface shows a typical wavy pattern as shown in Figure 11. Further, the size of the reinforced SiC_p_ is smaller than the spark gap and a part of the particle is partly bounded in the matrix material. The remaining protruded part is exposed to high temperatures in the spark gap. Figure 10, Figure 11 and Figure 12 reveal that the protruded portion of the SiC_p_ to get pushed further inside the molten matrix pool during the machining process. This is mainly due to the combined effect of impact of shock waves created by spark and mechanical moment by travelling wire electrode on the reinforced particles. These figures also show that all the SiC_p_ are intact with the machined surface and debonding of the particles from the matrix is not seen.

Further, it is evident from Figure 12 and Figure 13 that the machined surface shows the presence of micro-cracks and blow holes of very small size. These cracks are primarily formed due to the shrinkage of matrix material which occur by rapid and repetitive cooling of the molten pool by the circulating dielectric fluid. The presence of blow holes is mainly due to interdentritic segregation of alloying elements such as Mg, Si, etc. present in Al6061 alloy and rapid cooling of molten metal. The gas evolved during the sparking process in the dielectric medium also contributes to the formation of the blow holes on the machined surface.

### 3.5. Analysis of Variance (ANOVA) of MRR

The results of ANOVA of the MRR are shown in Table 4. The F-test is conducted on the ANOVA data at 95% confidence level to identify process parameters which are significantly affecting MRR. The F-values of the current, gap voltage, pulse-on time and pulse-off time are found to be higher than the critical values and hence their effect on MRR is significant. Further, the percentage contribution of each process parameter is calculated. The current is the most significant parameter that contributed maximum (27.39%) to variation of material removal rate followed by voltage (22.08%), pulse-off time (21.32%), pulse-on time (15.76%) and wire speed (12.94%).

### 3.6. Optimum of Process Parameters

The Taguchi method is adopted for the optimization of machining settings which produce maximum MRR while machining of Al6061-SiC_p_ composite by wire-EDM. Table 5 shows the average MRR obtained at different settings of process parameters. From the table, it is observed that the maximum MRR is achieved at the settings A_3_B_3_C_1_D_2_E_2_, i.e., current: level 3 (5A), pulse-on time: level 3 (40 µs), pulse-off time: 10 µs (level 1), drum/wire speed—level 3 (700 rpm) and voltage—90 V (level 2). The predicted MRR at this setting are 69.33 mg/min and 67.28 mg/min for samples with 4% and 8% SiC_p_, respectively. Further, the confirmation experiments are conducted, and the test results are shown in the Table 6. It is seen that MRR obtained by the experiments is in agreement with the predicted MRR. Further, the maximum change (delta) in MRR due to change in settings of different process parameters at different levels is also shown in the same table. Based on the delta values, the process parameters are ranked in the order of their influence as current: I, pulse-off time: II, pulse-on time: III, wire-speed: IV and voltage: V.

### 3.7. Regression Modelling of MRR

This section describes the details of developing the statistical model to predict the MRR for different settings of the process parameters which is possible in wire-EDM (Model: Concord/DK7732). A statistical model is developed to establish relationship of *I*: Current, *t_on_:* Pulse-on time, *t_off_*: Pulse-off time, *w_s_*: wire-speed, *I_v_*: voltage and the MRR. The main effect of these parameters is considered in the modelling. The generalized form of the regression model is given by Equation (9), and the developed model is given by Equation (10) for the samples with 4% and 8% SiC_p_. The coefficient of determination (𝑅^2^) for the equation is 73.6%.

(9) $$\begin{array}{l} y=c+k_{1}\times x_{1}+k_{2}\times x_{2}+.........+k_{n}\times x_{n} \end{array}$$

where,

*y*—dependent parameter;

*c*—model constant;

*x*_1_·······*x*_*n*_—control parameter;

*k*_1_·······*k*_*n*_—coefficients of control parameters.


(10) $$\mathrm{MRR}\;=-\;1.20+0.114\times \;I+0.00685\times \;t_{on}-0.0194\times \;t_{off}+0.000135\times \;w_{s}+0.0142\times \;Iv$$

**Figure 14:**
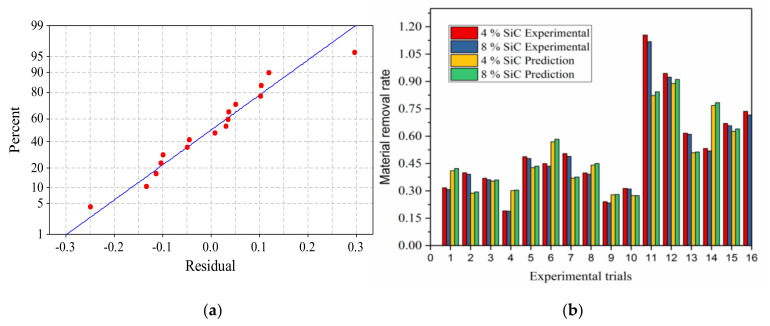
(**a**) Distribution of residuals. (**b**) Comparison of MRR predicted by model with experimental results.

The accuracy of predicting MRR by regression model is tested by conducting confirmation experiments within the range of operating parameters, that is, 3 ≤ I ≤ 6; 20 ≤ t_on_ ≤ 50; 10 ≤ t_off_ ≤ 25; 375 ≤ w_s_ ≤ 1400; 80 ≤ I_v_ ≤ 90. The distribution of residuals of the model is shown in Figure 14a which shows that the residuals are distributed around the line of fit linearly and hence confirms the adequacy test model linearity. Figure 14b shows the MRR predicted by the regression model and the experimentally obtained. It is observed that the actual MRR for the test sample with 4 wt. % and 8 wt. % are close to each other. The minimum and average predication error MRR is 4.45% and 20.46%, respectively. Standard deviation of error 2.19.

## 4. Conclusions

This study investigated the effect of parameters such as *I, t_on_, t_off_, w_s_, I_v_* on machining of 4 wt. % to 8 wt. % SiC_p_—Al6061 alloy by wire-EDM. These parameters are optimized to improve the MRR. The statistical model developed is useful for predicting the MRR for different settings of the process parameters. The optimized settings can be used to improve the productivity by increasing the MRR while machining of Al6061-SiC_p_ (up to 8 wt. %) alloy by wire-EDM industries. The following conclusions are drawn based on the results obtained.

- The current, *t_on_, t_off_*, wire speed and voltage showed significant influence on MRR. The MRR increased with increase in current, *t_on_*, wire speed and voltage, but it decreased with increasing the *t_off_* and also *w_s_* beyond 700 rpm.
- The current contributed to maximum (27.39%) variation of MRR followed by voltage (22.08%), *t_on_* (21.32%), *t_off_* (15.76%) and *w_s_* (12.94%).
- The optimum settings which produced highest MRR (65.21 mg/min and 62.41 mg/min for samples with 4% and 8% SiC_p_) are current −5 A, *t_on_*—40 µs, *t_off_*—10 µs, *w_s_*—700 rpm and voltage—90 V.
- Addition of SiC_p_ up to 8% in the Al6061 matrix did not show significant difference in MRR. However, a marginal decrease of MRR is observed with increase in SiC_p_ content.
- The machined surface showed the formation of craters and recast layers. Particularly on the recast layers, the cracks and blow holes are seen which affects the surface finish.
- The statistical model (average 𝑅^2^—73.65%) is developed to predict the MRR which can effectively be used to estimate the MRR in wire-EDM of Al6061 with SiC_p_ up to 8 wt. % within the operating range (3 A ≤ *I* ≤ 6 A; 20 µs ≤ *t_on_* ≤ 50 µs; 10 µs ≤ *t_off_* ≤ 25 µs; 375 rpm ≤ *w_s_* ≤ 1400 rpm; 80 V ≤ *I_v_* ≤ 90 V).
- The minimum and average prediction error of MRR from the proposed regression model is 4.45% and 20.46%, respectively. An attempt can be made to reduce these errors by using advanced methods such as firefly, genetic algorithms and other nature-inspired optimization methods and a comparative study of the effectiveness of optimization and predication accuracy can be made.

## Figures and Tables

**Table 1:** Composition (%) of Al6061.

Si	Fe	Cu	Mn	Mg	Cr	Zn	Ti	Others	Al
0.40–0.80	0.70	0.15–0.40	0.15	0.80–1.20	0.04–0.35	0.25	0.15	0.05	Rest

**Table 2:** Wire-EDM process parameters and settings.

Parameters	Unit	Code	1	2	3	4
Current	A	A	3	4	5	6
Pulse-on time	µs	B	20	30	40	50
Pulse-off time	µs	C	10	15	20	25
wire speed	RPM	D	175	350	700	1400
voltage	V	E	80	90	-	-

**Table 3:** The experimental design and corresponding average MRR.

Trial No	Settings of the Process Parameters					MRR	
	I	t_on_	t_off_	W_s_	V	4%	8%
1	3	20	10	1400	80	0.317	0.307
2	3	30	15	700	80	0.399	0.391
3	3	40	20	350	90	0.369	0.361
4	3	50	25	175	90	0.190	0.188
5	4	20	15	350	90	0.487	0.477
6	4	30	10	175	90	0.449	0.435
7	4	40	25	1400	80	0.504	0.489
8	4	50	20	700	80	0.398	0.391
9	5	20	20	175	80	0.240	0.234
10	5	30	25	350	80	0.313	0.310
11	5	40	10	700	90	1.154	1.118
12	5	50	15	1400	90	0.944	0.923
13	6	20	25	700	90	0.616	0.610
14	6	30	20	1400	90	0.532	0.519
15	6	40	15	175	80	0.670	0.657
16	6	50	10	350	80	0.736	0.716

**Table 4:** Analysis of variance for MRR.

Source	DF	4% Sic			8% Sic		
		Seq SS	Adj MS	F	Seq SS	Adj MS	F
Current	3	0.3004	0.1001	57.57	0.3148	0.1049	50.84
Pulse-on time	3	0.1729	0.0576	33.13	0.1854	0.0618	29.95
Pulse-off time	3	0.2338	0.0779	44.81	0.2524	0.0841	40.77
Wire speed	3	0.1419	0.0473	27.19	0.1492	0.0497	24.10
Voltage	1	0.0807	0.0807	46.41	0.0844	0.0844	40.92
Residual Error	2	0.0034	0.0017		0.3148	0.1049	
Total	15	0.9334					

DF: degree of freedom, MS: mean square, SS: sum of square.

**Table 5:** The average MRR.

Level	Current	Pulse-On Time	Pulse-Off Time	Wire Speed	Voltage
**4% SiC_p_**					
1	0.318	0.415	0.663	0.574	0.447
2	0.459	0.423	0.625	0.641	0.592
3	0.662	0.674	0.384	0.476	-
4	0.638	0.567	0.405	0.387	-
Delta	0.344	0.259	0.279	0.254	0.145
**8% SiC_p_**					
1	0.312	0.407	0.643	0.378	0.436
2	0.448	0.413	0.612	0.466	0.578
3	0.646	0.656	0.376	0.627	-
4	0.625	0.554	0.399	0.559	-
Delta	0.334	0.249	0.267	0.249	0.142

**Table 6:** The predicted and experimental MRR (mg/min) at optimum conditions.

Trial No	4%			8%		
	Predicted	Experimental	Error	Predicted	Experimental	Error
1	69.33	65.66	5.30%	67.28	62.52	7.08%
2	69.33	64.57	6.87%	67.28	61.96	7.90%
3	69.33	66.15	4.58%	67.28	63.30	5.91%
4	69.33	64.46	7.00%	67.28	61.88	8.02%

## Data Availability

The data presented in this study are available on request.

## Nomenclature

ANOVA	Analysis of variance
DF	Degree of freedom
E_s_	Energy supplied
F-Test	Fishers test
I	Current
I_v_	Input voltage
MRR	Material removal rate
MWCNTs	Multiwalled carbon nanotubes
MS	Mean square
q_R_	Maximum heat intensity
R	Radial distance from the spark axis
r_sp_	Plasma channel radius
SEM	Scanning electron microscopy.
SS	Sum of square
SiC_p_	Silicon carbide particles
T_on_	Pulse-on time
T_off_	Pulse-off time
Γ	Volume of material melted
Wire-EDM	Wire-electrical discharge machining
W_s_	Wire speed
w_i_	Weight before the machining
w_f_	Weight after the machining
W_m_	Energy utilized by the material
